# Determinant genetic markers of semen quality in livestock

**DOI:** 10.3389/fendo.2024.1456305

**Published:** 2024-10-04

**Authors:** Muhammad Zahoor Khan, Wenting Chen, Saima Naz, Xiaotong Liu, Huili Liang, Yinghui Chen, Xiyan Kou, Yihong Liu, Iqra Ashraf, Ying Han, Yongdong Peng, Changfa Wang, Muhammad Zahoor

**Affiliations:** ^1^ Liaocheng Research Institute of Donkey High-Efficiency Breeding and Ecological Feeding, Liaocheng University, Liaocheng, China; ^2^ Department of Zoology, Government Sadiq College Women University, Bahawalpur, Pakistan; ^3^ Department of Molecular Medicine, Institute of Basic Medical Sciences, University of Oslo, Oslo, Norway

**Keywords:** livestock, selective breeding, genetic markers, semen quality traits, reproductive efficiency

## Abstract

The reproductive efficiency of livestock is crucial for agricultural productivity and economic sustainability. One critical factor in successful fertilization and the viability of offspring is the quality of semen. Poor semen quality, especially in frozen-thawed semen used in artificial insemination (AI) have been shown to influence conception outcomes, resulting a negative impact on livestock production. Recent advancements in genetic research have identified specific markers linked to semen quality traits in various livestock species, such as cattle, sheep, goats, pigs, buffalo, and equines. These genetic markers are essential in screening males for breeding suitability, which in turn enhances selective breeding programs. Understanding these markers is crucial for improving reproductive performance and increasing productivity in livestock populations. This review offers a comprehensive overview of the genetic markers associated with semen quality in key livestock. It explores the underlying genetic mechanisms and their practical implications in animal breeding and management. The review underscores the importance of integrating genetic insights into breeding strategies to optimize reproductive efficiency and ensure the sustainable development of livestock industries.

## 1 Introduction

The reproductive efficiency of livestock is a vital factor that significantly impacts agricultural productivity and economic sustainability. Semen quality is particularly crucial in determining successful fertilization and the resulting offspring’s outcomes (1, 2). Sperm disorders in frozen–thawed semen, widely used in artificial insemination (AI) technology, confer a risk of impaired fertility in livestock. In addition, poor quality may lead to male infertility (3, 4). The quality of semen plays a crucial role in the reproductive success of livestock by directly impacting fertility rates, genetic diversity, and overall herd productivity. The efficiency of breeding programs and the profitability of livestock farming depend on the ability to produce offspring with desirable traits.

The assessment of semen quality traditionally relies on conventional methods, which evaluate sperm motility, morphology, concentration, and ejaculate volume. Over time, there have been significant improvements in this assessment, allowing for a more comprehensive evaluation of sperm quality and fertility parameters (5). However, these methods do not consider the molecular characteristics of sperm cells, such as DNA integrity, oxidative status, or the presence of essential sperm proteins (6). This limitation can hinder the identification of molecular causes of subfertility. Several tests have been developed to predict semen quality, but a single, highly reliable test is not yet available (7, 8). Genetic markers provide valuable information about the genetic factors that determine semen quality (9, 10). They enable accurate predictions and early detection of problems before physical symptoms appear. These markers are crucial for monitoring how semen quality traits are inherited across generations, making them extremely valuable for breeding programs. By using genetic markers, we can make better choices when selecting individuals with the best traits (8, 11, 12). This also helps us to understand the genetic basis of semen quality and reduces the impact of environmental factors. As a result, the assessment of semen quality becomes more stable and reliable compared to traditional methods that rely solely on physical characteristics (9, 13).

Recent advancements in genetic research have enabled the identification of specific genetic markers associated with semen quality traits in various domestic livestock species (14–16), including sheep (17), goats (18), pigs (19), cattle (20), buffalo (21) and equines (22). In addition, these genetic markers also play a key role in screening the suitability of male for breeding purposes (23). Thus, understanding these genetic markers is essential for enhancing selective breeding programs, improving reproductive performance, and ultimately increasing the productivity of livestock populations. This review study aims to provide a comprehensive review of the genetic markers linked to semen quality in livestock, exploring the underlying genetic mechanisms and their practical implications in animal breeding and management.

## 2 Methodology

This review provides a comprehensive overview of the genes associated with semen quality traits across various livestock species, including buffalo, cattle, equine, pigs, sheep, and goats. The majority of the data considered in this review were derived from publications spanning from 2010 to 2024, with an additional inclusion of data from ten articles published between 2003 and 2010. The literature search was conducted using databases such as Google Scholar, X-MOL, PubMed, and Scopus. Only articles published in English and indexed in SCI journals were considered. The search employed keywords such as ‘pigs,’ ‘cattle,’ ‘buffalo,’ ‘horses,’ ‘sheep,’ ‘goats,’ ‘semen quality traits,’ and ‘genetic markers associated with semen quality traits. Notably, data from book chapters, conference proceedings, and letters to the editor were excluded from this review. Finally, the DAVID online tool (https://david.ncifcrf.gov/tools.jsp) was utilized to identify the biological processes and signaling pathways of genes associated with semen quality traits in livestock.

## 3 Genetic markers associated with semen quality phenotypic traits in cattle bulls

Various approaches, such as genome-wide association studies (GWAS), transcriptomic analysis, and the candidate gene method, have been utilized to screen genes associated with semen quality traits in cattle bulls (24, 25). GWAS analysis involves evaluating genomes from multiple phenotypes to identify genetic markers that can predict the presence of a trait. Once these markers are identified, they can be used to understand how genes contribute to the traits. For example, a study found that *DYRK1A, TEC*, and *TXK* were associated with sperm motility based on GWAS analysis (10). Another study documented the association of *GALNTL6, HMGB2, ADAM29, PRMT6, SCAPER, EDC3*, and *LIN28B* with spermatogenesis, ejaculate volume, sperm concentration, and sperm motility (26). Similarly, GWAS analysis has led to the discovery of several genes associated with spermatogenesis, total sperm motility, and progressive sperm motility in Italian Holstein bulls (27), as shown in **Table 1**.

**Table 1:** Genetic markers associated with semen quality phenotypic traits in cattle bull.

Genes	Associated with semen quality phenotypic traits
*LPCAT4, CACNB2, IGFBP3, STEAP1, POU6F2, PPP1CB, AQP7*	Acrosomal integrity
*CDF9, MARCH1, WDR19, SLOICI, ST7, DOP1B, CFAF9, INHBA, ADAMTS1*	Sperm motility, semen volume, sperm count, sperm concentration, sperm head, sperm integrity, sperm tail abnormalities, and percentage of abnormal sperm traits.
*GART, ESR1, MAP2K5, ZFYVE26, RAD51B, TTC29, SPADH2, GPR26, FGFR2*	Damaged sperm tails and cell necks
*OPN, TNP1, TNP2, PSMB5, PRMT5, ACTB, NPC1, FSCN1, NR5A2, IQCG, LHX8, DMRT1, HIBADH, PCK1, KIT, CDH1, PRM1, PRM2, DAZL, PPIA, INRA, SPAG11, PRNP, CAPN1*	Ejaculate volume and sperm motility
*SPATA7, PI4KB, DPY19L2*	Spermatogenesis, sperm capacitation and acrosome formation
*UBE2D3, CASP3, HSFY2*	Percentage progressive motile spermatozoa
*PRKCB, CFTR, IGF1R, SRD5A2, CATSPER1*	Poor sperm motility
*MAP3K1, VIP, SOD2, TCP1, PACRG, SPEF2*	Scrotal circumference, sperm motility and male fertility
*DCP1A, PRKCD, PHF7, TLR9*	Semen volume and total number of sperm
*ETNK1, PDE3A, PDGFRB, CSF1R, WT1, DSCAML1, RUNX2, FSHR, INHA, PRL, PLCz, TSPY*	Semen volume per ejaculate, initial sperm motility, sperm concentration per ejaculate, number of sperm per ejaculate, number of motile sperm per ejaculate
*HDAC9, ID2, GSTT1, GSTM1, CDK5, NOS3, PRL, CD9, RAPD*	Sperm concentration and motility
*KAT8, CKB, TDRD9*	Sperm development and motility
*ORC4, EPC2, MBD5, CFAP58*	Sperm head abnormalities
*SGMS2, TET2, GSTCD*	Sperm membrane integrity
*DYRK1A, TEC TXK, FHDC1, PARK2, GALNT13, PRM1, PRM2, BM1500, UMN2008, INHBE, INHBC, HELB, INCENP, Tf, PSMA1, SNCAIP, RPL31, PRKCE, PAPSS2, PLP1, R1G7, LHR, GnRHR*	Sperm motility
*SGMS2, TET2, GSTCD*	Sperm plasma membrane integrity
*TUBB2C, HSP10, HXK1, SOD1, AQP7*	Sperm viability
*GALNTL6, HMGB2, ADAM29, PRMT6, SCAPER, EDC3, LIN28B, ZNF280B, SLC26A2, DMXL1, OR52A1, MACROD2, REV1, JAKMIP1, PPP1R11, HSPA4, MORC1, SPATA21, GSTA4, FSCN3, EFHC1, CSNK1G2, EPHA2, FAM9B, TBL1X, PIH1D3*	Spermatogenesis and male fertility
*BMP2, NGF*	Semen mitochondrial membrane potential

The information on genes and their association with semen quality phenotypic traits in bulls has been obtained from previous studies (10, 14, 24, 26, 36–80).

Recent studies have documented the association of *PRM1, STK35*, and *IFT27* (28), *FOXO4, FOXP3, GATA1, CYP27B1, EBP, KDM5C, LRRK2*, and *PME* (29), and *MARCH1* (14) with semen quality traits and bull fertility. Another study reported the upregulation of *SPADH2, TIMP-2, PLA2G7, OAZ3, GPx4*, and *GSTM3* in bulls with reduced sperm motility and fertility (30). In contrast, the levels of *caltrin* and *ADM* were low in bulls with high ejaculate rejection rates, indicating a strong link between these proteins and sperm motility (30). Furthermore, *FBXO39* was found to be differentially expressed in sperm cells and seminal plasma, showing a strong correlation with sperm motility in bulls (20). Recently, research compared the genetic marker profiles of seminal plasma from breeding bulls producing good and poor-quality semen (31). Consistently, a study conducted proteomic analysis of seminal plasma and found that *CCL2, UQCRC2*, and SAA1 were upregulated in the seminal plasma of poor-quality semen and negatively associated with sperm functions (32). Furthermore, *NGF, EEF1A2, COL1A2, IZUMO4, PRSS1, COL1A1, WFDC2, COL1A1, COL2A1, COL1A2, SPP1*, and *PDGFA* were found to have a positive effect on sperm function and were downregulated in the seminal plasma of poor-quality semen.

Interestingly, the association of *TPT1, BOLA-DRA, CD74, RPS17, RPS28, RPS29, RPL14, RPL13*, and *RPS27A* with sperm functionality, survival, oxidative stress, and bull fertility has been discovered (33). Furthermore, it has been revealed that *POU4F2, GRIK1, NEDD4, FOXF1, RAD51B, WNT4, WNT5A, RIMS1* and *PPP3CA* were key genes associated with sperm head and tail disorders in cryopreserved semen of bulls (34). Consistently, another study found that polymorphisms in *FSHR*, *INHA, INHAB, TNP2*, and *SPEF2* genes were significantly correlated with doublet ejaculate volume, sperm concentration, progressive motility, and total number of spermatozoa in bulls (35). These genes were found to be associated with sperm structural integrity, cellular communication, and DNA repair, all of which are important for spermatogenesis and sperm function.

## 4 Genetic markers associated with semen quality phenotypic in buffalo bull

Improving the quality of buffalo semen is a crucial focus in the fields of livestock genetics and reproductive biotechnology. Recent advancements have identified genetic markers in buffalo bulls that show promise in enhancing semen quality. For instance, one study found that variations in the Leptin gene are linked to higher progressive motility (PR), increased sperm concentration, total sperm count, and elevated levels of *LH* and testosterone hormones (81). Another research study identified significant associations between the expressions of *MAPK3, RPL36AL, EXT2, RPS27A, RPS18*, and *RPS28* with progressive motility, acrosome integrity, functional membrane integrity, and overall fertility rate (82). Similarly, GWAS analysis discovered several genes, including *TEKT2, SPEM1, PRM3, EQTN, PLCZ1, SPESP1, SPACA1, TNP1*, and *YBX2*, that may influence sperm motility as well as the structural and functional membrane integrities of sperm (83). Furthermore, the association between *RPL10, ZCCHC13, AKAP4, TSPAN6, RPL10*, and *RPS4X* and sperm motility has been well established (84). Another study highlighted a positive correlation between *GnRHR* and percentages of sperm motility, sperm concentration, and live sperm count (85). Higher expression levels of *PDZD8, GTF2F2, ZNF397, KIZ, LOH12CR1, ACRBP, PRSS37, CYP11B2, F13A1* and *SPO11* were found in high-fertile spermatozoa, whereas overexpression of *MT1A, ATP5F1, CS, TCRB, PRODH2, HARS, IDH3A, SRPK3, TUBB2B, GPR4, PMP2, CTSL1, TPPP2* and *EGFL6* were reported in low-fertile spermatozoa (86). For easy reference, we have summarized the research progress on genes associated with semen quality traits in buffalo bulls in **Table 2**.

**Table 2:** Genetic markers associated with semen quality phenotypic traits in buffalo bull.

Genes	Associated with semen quality phenotypic traits
*GnRHR*	Sperm concentration, post-thaw sperm motility, sperm abnormality and sperm ejaculate volume
*LHβ*	Sperm concentration (million/mL), percent mass motility, acrosome and membrane integrity
*SFRP1, STXBP4, BCR, PSMG4, ARSG, ATP11A, RXRA*	Spermatogenesis
*SPINK2, NEDD8, YBX2*	Spermatogenesis and sperm motility
*PRM1, AKAP3*	Sperm motility
*R2T11, OR10S1*	Spermatogenesis
*YBX1, ORAI3, TFAP2C*	Sperm motility and spermatogenesis
*VAMP4, APOC3*	Sperm maturation and capacitation
*PHB, CAPZB, TEKT2*	Sperm motility
*OPN*	Sperm concentration, sperm motility and the lowest total number of sperm pathologies
*IGF-1*	Spermatozoa motility

The data on genes and their association with phenotypic traits of semen quality in buffalo bulls has been collected from previous studies (85–99).

## 5 Genetic markers associated with semen quality phenotypic traits in buck and ram

Fertility is essential for the overall reproductive success of sheep and goats, playing a vital role in the small ruminant industry. Similar to other livestock, semen quality is also crucial in sheep and goats for successful conception. Significant research has been conducted on the screening of genes and their association with semen quality traits in bucks and rams, including volume, gross motility, concentration, percent post-thaw motility, number of spermatozoa, and sperm abnormalities (100, 101). Previous GWAS studies consistently identified several candidate genes related to semen quality traits in sheep (102, 103) and goats (104). In order to improve clarity and facilitate understanding, we have provided a summary of studies on genes associated with semen quality traits in **Table 3**.

**Table 3:** Genetic markers associated with semen quality phenotypic traits in buck and ram.

Genes	Associated with semen quality phenotypic traits
*ARHGEF38, TIGD2, PCDH7 SCAPER, PSMA4, CABLES1*	Ejaculate volume
*CCSER1, KCNIP4, GBA3 STIM2, OCIAD1, HOPX, LOC101110593*	Sperm motility and membrane integrity
*IL7R, CFB*	Sperm motility, cell growth, homeostasis of number of cells, regulation of the immune reaction
*DOCK2, CPLANE1, SLC9C1, GRM8, PAQR3, BMP2K, NCALD, CMIP, SORD, SH2B1, NT5E, PARM1, FSHβ, CUL9, DSCAML1, FSHβ, LHβ*	Sperm motility and spermatogenesis
*MTNR1A and CYP19, SMAD2, BMP1R, PPP3CA*	Sperm volume, sperm concentration, total spermatozoa per ejaculate, sperm motility, and testicular sizes
*ITGA4/6/9, TGFB2, TGFBR1, TGFBR2, JAM3, SMAD3, NDRG1, FSCN3, CYP26B1, Leptin, RAI14*	Spermatogenesis
*SREBP1, ELOVL2*	Spermatogenesis and remodeling of the membranes of developing germ cells
*SOX9, BCL2, HDC, GGT5, ZNF280BY*	Spermatogenesis and testicular development
*DPY19L2, RNF17, TDRD5, SUN5, MEIOC, KLHL10, PLD6, TNP1, TSS6, SPAG6, CAPZA3, SPAG11*	Spermatogenesis, spermatid development, and flagellated sperm motility
*ODF3, ZPBP1, INSL3, AMH, INHBA, COL1A1, COL1A2, INHA, PDGFA, IGF1, DNAH17, SPATA4, CIB4*	Spermatogenesis, sperm motility, structural integrity of sperm tails, testis development, size and male fertility

The data on genes and their correlation with phenotypic traits of semen quality in bucks and rams has been collected from previous sources (17, 18, 99–116).

## 6 Genetic markers associated with semen quality phenotypic traits in boars

The use of AI in swine production allows for the selection of boars based on their desirable production traits. However, AI heightens the importance of each boar’s reproductive performance, necessitating the evaluation of semen samples for their fertilization potential at boar stations (117). The pig industry aims to maximize the number of insemination doses produced from each boar ejaculate, which requires boars to produce high-quality semen characterized by high motility, progressive motility, and low levels of morphological defects, in large quantities (a high number of sperm cells per ejaculate) (118).

Spermatogenesis and fertilization are complex processes regulated by numerous genes. For instance, *ACTN1* and *ACTG2* significantly impact semen volume per ejaculate and sperm motility (117). Lin et al. identified several candidate genes, including gonadotropin-releasing hormone receptor (*GNRHR*), prolactin (*PRL*), prolactin receptor (*PRLR*), follicle-stimulating hormone beta (*FSHB*), luteinizing hormone beta (*LHB*), follistatin (*FST*), inhibin alpha (*INHA*), inhibin beta A (*INHBA*), retinol-binding protein 4 (*RBP4*), androgen receptor (*AR*), relaxin (*RLN*), acrosin (*ACR*), osteopontin (*OPN*), and β-actin (*ACTB*) that were associated with sperm quality traits such as sperm concentration, motility, semen volume per ejaculate, plasma droplets rate, and abnormal sperm rate (119–121). Further studies have highlighted the roles of phospholipase C zeta (*PLCz*), cyclooxygenase isoenzyme type 2 (*COX-2*) (122), and cluster-of-differentiation antigen 9 (*CD9*) (123), along with estrogen receptor 1 (*ESR1*) and *ESR2*, in spermatogenesis and semen quality traits like sperm concentration, motility, semen volume, plasma droplet rate, and abnormal spermatozoa rate (124, 125). *TEX14* has been associated with spermatogenic arrest and subsequent infertility in boars (126). Similarly, *TK17b* and *HECW2* are linked to severe defects in sperm acrosome and chromatin, causing infertility (127). Genes such as *EGF, PTGS2*, and *PRLR* have been positively correlated with semen volume per ejaculate, sperm motility, percentage of normal sperm, percentage of sperm with proximal plasma droplets, and total sperm count per ejaculate (128). The *DAZL* gene has been associated with lower sperm motility and concentration in boars (129). Polymorphisms in genes like *CD9* (g.358A>T), *ESR1* (g.35756T>C), and *PLCz* (g.158T>C) have been linked to sperm motility (130–132). Additionally, *RAMP2* and *GIMAP6* were identified through RNA-seq analysis and found to be associated with sperm DNA fragmentation in boars (133). Similarly, another study revealed through RNA-analysis that genes such as *FOS, NFATC3, EAF2, BAMBI, PTPRU, PTPN2, ND6*, *ACADM*, and *FGF-14* were associated with spermatogenesis, energy metabolism and poor semen freezability (134).

Genome-wide association studies (GWAS) have been used to identify genetic markers associated with semen quality traits in boars. A study reported the link of mitochondrial methionyl-tRNA formyltransferase (*MTFMT*) is associated with sperm motility (135). Similarly, another research identified *PLA2G4A, PTGS2*, and *HPGDS* as markers associated with motility, progressive motility, the number of sperm cells per ejaculate, and total morphological defects, all using GWAS (136). Accordingly, the association of *PRMT6, Sox5, PEX10, SIRPA*, and *SIRPG* with oligozoospermia in Han Chinese Population has been explored (137). Furthermore, a GWAS analysis revealed several key genes and their association with semen quality traits and spermiogenesis including *TDRD5, QSOX1, BLK, TIMP3, THRA, CSF3*, and *ZPBP1* with number of sperm cells, *PPP2R2B, NEK2, NDRG, ADAM7, SKP2*, and *RNASET2* with sperm motility; *SH2B1, BLK, LAMB1, VPS4A, SPAG9, LCN2*, and *DNM1* with sperm progressive motility, *GHR, SELENOP, SLC16A5, SLC9A3R1*, and *DNAI2* with total morphological abnormalities (138). Interestingly, genetic markers have also been identified through GWAS analysis that were associated sperm morphology, deformities and semen qualities (139). Several other genes such *CHD2, KATNAL2, SLC14A2, ABCA1, PRM1, OAZ3, DNAJB8, TPPP2, IQCJ, ACTR2, HARS* and *TNP* have been found to be correlated with percentage of head and neck abnormalities, abnormal acrosomes and motile spermatozoa (140). To facilitate understanding, a summary of studies on genes associated with semen quality traits is provided in the accompanying **Table 4**.

**Table 4:** Genetic markers associated with semen quality phenotypic traits in boar.

Genes	Associated with semen quality phenotypic traits
*FOXL3, GPER1, PDGFA, PPP1CC, CSNK1G2, PSMF1, PRKAR1B, SUN1, TSPO, SPAG6, H2AFZ, RNF4, NR4A1*	Spermatogenesis, sperm motility and ejaculate volume
*CEP78, DNAAF5, KCNA, GPER1, CRISP3, Kiss1, C7H15orf39, NOS2, PTBP2, STRA8*	Sperm motility
*PTGES, SFRP1, SPP1, PLA2G4E, KCNJ5, PTGS2, HCN1, DAZL, BCAS2*	Spermatogenesis and testicular development
*ZSWIM7, TEKT3, UBB, EIF2B2, MLH3, CCDC70*	Sperm rate and count
*TXNRD1, HSPA4L, ATP1B1*	Spermatogenesis, sperm integrity and motility
*ESR, FSHB, PRLR, STK35, IFT27, HSPD1*	Sperm ejaculate volume, sperm motility and sperm concentration
*B9D2, PAFAH1B3, TMEM145, CIC*	Sperm concentration
*WWC2, CDKN2AIP, ING2, TRAPPC11, STOX2, PELO*	Semen volume
*SMAD1, NF-1, FOXMI, RXRA, STAT4, BAMBI, RAB33B, CKS2, LARS2, SLC25A16, ACADM, CPT2*	Sperm motility and membrane integrity and spermatogenesis
*SCLT1, MAP3K20, MS4A2, ROBO1 APPL1, PLBD1, FBXO16, EML5, RAB3C, OXSR1, PRICKLE1*	Sperm motility and plasma membrane integrity of spermatozoa
*HOOK1, ARSA, SYCE3, SOD3, GMNN, RBPJ, STIL, FGF1*	Sperm coiled tail and sperm deformities
*FGF1, ADIPOR1, ARPC5, FGFR3, PANX1, IZUMO1R, ANKRD49, GAL*	Sperm bent tail
*NSF, WNT3, WNT9B, LYZL6, FGFR1OP, RNASET2, FYN, LRRC6, EPC1, DICER1, FNDC3A, PFN1*	Sperm proximal droplet
*OMA1, PFN1, PELP1, BMP2, GPR18, TM9SF2, SPIN1*	Distal midpiece reflex
*ARSA, SYCE3, MOV10L1, CBR1, KDM6B, TP53, PTBP2, UBR7, KIF18A, ADAM15, FAAH, TEKT3, SRD5A1*	Distal droplet
*CHD2, KATNAL2, SLC14A2, ABCA1, PRM1, OAZ3, DNAJB8, TPPP2, TNP1, IQCJ, ACTR2, HARS*	Sperm motility and sperm morphology

The information on genes and their association with phenotypic traits related to semen quality in boars has been collected from previous studies (9, 19, 138–158).

## 7 Genetic markers associated with semen quality phenotypic traits in equine

Genetic factors are a major contributor to the wide range of semen quality observed in different horse populations (159–161). This variability has a significant impact on breeding success and reproductive efficacy in horses. Genetic traits influence important parameters like sperm motility, morphology, and overall viability, which are essential for successful fertilization. Recent GWAS analysis have identified specific genes associated with seminal traits, such as sperm concentration and motility (162, 163). For example, studies have highlighted the role of cysteine-rich secretory proteins (*CRISP1, CRISP2, CRISP3*), as well as other genes like *SIRT1, PGK2, CCT8, SOD1*, and *GLIPR1L1*, which have been linked to important semen quality traits (164–167). These genes play crucial roles in the structure and function of sperm cells, influencing their ability to fertilize an egg. Further GWAS research has discovered associations between additional genes, such as *NME8, OR2AP1*, and OR6C4, and sperm motility in stallions (22). The significance of these findings lies in the potential use of these genetic markers in selective breeding programs to improve reproductive outcomes in horses. Marker-based approaches using microsatellites have also provided insight into the genetic basis of semen quality. Variants within candidate genes like *SPATA1, PRLR, ACE, FKBP6, SP17, PLCz1*, and *FSHB* have been linked to sperm motility, which directly impacts the pregnancy rate per cycle, especially in German Warmblood horses (168–173). These genes are involved in critical processes such as sperm-egg fusion and the acrosome reaction, highlighting their importance in reproductive success. Furthermore, a recent study identified the gene *SCN8A*, associated with sperm motility. *SCN8A* encodes a sodium channel found in the flagellum and around the neck of mammalian spermatozoa, suggesting its role in regulating motility (174). Overall, this suggests that genetic variations in these genes may influence semen quality by affecting sperm development, survival in the reproductive tract, or capacitation and acrosome reaction.

## 8 Identifying key biological functions processes and pathways in genes linked to semen quality in livestock

In this review, we used the DAVID online software (175, 176) to analyze the pathways and functions of genes related to semen quality in livestock. Among the genes analyzed, we focused on those associated with hormonal regulation and receptor activity, including *GnRHR, LHR, LHβ, FSHβ, ESR1, ESR, PRLR, INHBA, INHA, INHBC, INHBE, MAD2, SMAD3, TGFB2, TGFBR1, TGFBR2*, and *FSHR.* Our analysis revealed their involvement in several key signaling pathways: the transforming growth factor-beta (TGF-β) signaling pathway (bta04350), prolactin signaling pathway (bta04917), cAMP signaling pathway (bta04024), and Hippo signaling pathway (bta04390).

Consistent with our findings, the literature suggests that components of the Hippo signaling pathway play a critical role in spermatogenesis and sexual maturity in male reproductive tracts of Hu sheep (177). Additionally, disruptions in Hippo signaling have been linked to sperm morphological abnormalities and infertility in patients with autosomal dominant polycystic kidney disease (178). The TGF-β signaling pathway is crucial for testis development and spermatogenesis and is implicated in maintaining male tract homeostasis and function (179). Notably, studies have shown that the absence of *IGF1* in sperm plasma membranes correlates with infertility (180), and the presence of *TGFβ1* and *TGFβ2* in porcine seminal plasma is associated with semen quality (181). Furthermore, *TGF-β* has been reported to modulate the immune environment of the female genital tract post-semen delivery during mating or artificial insemination (182). The cAMP signaling pathway is identified as a pivotal mechanism in gamete development, sperm capacitation, and fertilization, and it is targeted in infertility therapies (183, 184). Its role is further evidenced in regulating sperm motility in stallions (185) and has been implicated in affecting sperm motility in dairy goats via the alkaline dilution effect (186). The essential role of the prolactin signaling pathway is also underscored in our findings.

**Figure 1:**
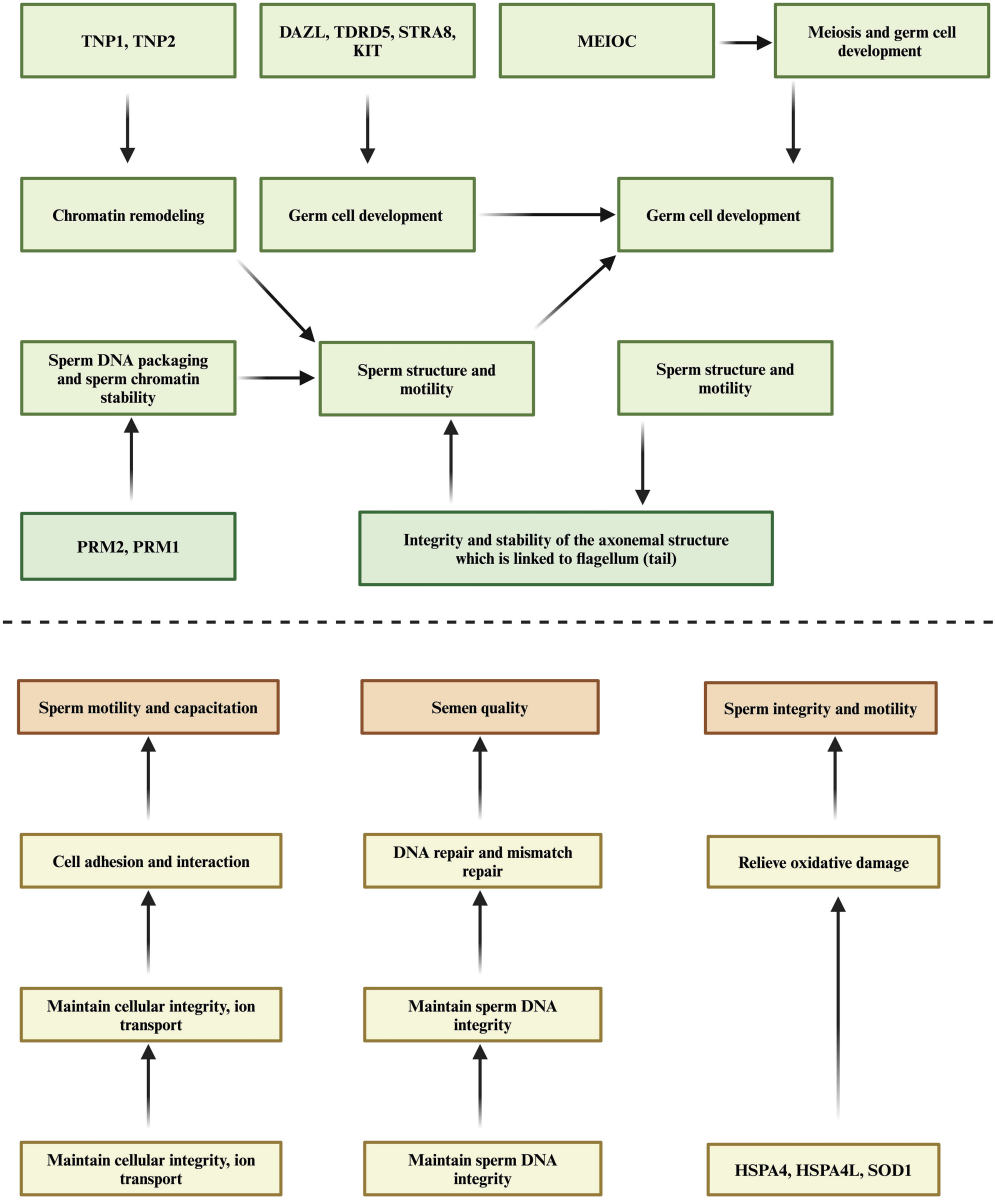
Schematic representation of genes involved in important biological processes and their associations with semen quality. This figure illustrates the relationships between various genes and their roles in biological processes such as sperm maturation, DNA integrity, spermatogenesis, oxidative stress response, and sperm motility. Please note that this figure is based on speculative information rather than validated data, and the depicted relationships should be interpreted with caution.

Further analysis revealed that genes involved in energy metabolism, mitochondrial function spermatogenesis, sperm development, sperm motility and structure (*TEKT, TNP, PRM1, TNP, CDH, HSPA, DAZL, STRA, DPY19L2, KIT, MEIOC* and *KLHL10* etc.), significantly regulate other signaling pathways, including MAPK (bta04010), cytoskeleton in muscle cells (bta04820), and PI3K-Akt signaling pathway (bta04151). The biological functions of these genes are summarized in **Table 5** and **Figure 1**. Additionally, genes implicated in apoptosis (*CATSPER1, BCL2, BAX* and *CASP3*) influence pathways such as Apoptosis - multiple species (bta04215) and p53 signaling pathway (bta04115). The p53 signaling pathway is noted for its role in maintaining semen quality by ensuring the quantity and quality of mature sperm and regulating reproductive processes such as genomic integrity and germ cell pools (187). Moreover, the SPATA18-P53 pathway is crucial for controlling mitochondrial quality by eliminating oxidative proteins, as oxidative stress can adversely affect sperm motility and quality by upregulating *p53* expression (188). Lastly, genes related to the antioxidant response (*SOD1, SOD2*) significantly regulate the Peroxisome signaling pathway (bta04146). Peroxisome proliferator-activated receptor gamma (*PPARγ*) is suggested to link lipid metabolism with overall reproductive functions, providing essential energy from glucose and fat metabolism for sperm physiology and influencing male fertility (189, 190).

**Table 5:** Gene ontology (GO) analysis of biological processes linked to semen quality genes.

Biological functions	Genes
GO:0007286~spermatid development	*PRM2, DPY19L2, KIT, MEIOC, KLHL10, TNP1, TDRD5*
GO:0007283~spermatogenesis	*DAZL, STRA8, PRM2, PRM1, KIT, TNP2, TNP1, TDRD5, SUN5*
GO:0035092~sperm DNA condensation	*PRM1, TNP2, TNP1*
GO:0030317~flagellated sperm motility	*TEKT2, TEKT3, TNP1*
GO:0010954~positive regulation of protein processing	*TNP2, TNP1*
GO:0030261~chromosome condensation	*PRM2, PRM1*
GO:0060294~cilium movement involved in cell motility	*TEKT2, TEKT3*
GO:0007155~cell adhesion	*VCAM1, ATP1B1, JAM3, ICAM1*
GO:0072659~protein localization to plasma membrane	*CDH2, CDH1, ATP1B1*
GO:0098609~cell-cell adhesion	*VCAM1, CDH2, ICAM1*
GO:0006298~mismatch repair	*MSH2, MLH3*
GO:0007416~synapse assembly	*CDH2, CDH1*
GO:0044331~cell-cell adhesion mediated by cadherin	*CDH2, CDH1*
GO:0016339~calcium-dependent cell-cell adhesion via plasma membrane cell adhesion molecules	*CDH2, CDH1*
GO:0000902~cell morphogenesis	*CDH2, CDH1*
GO:0006457~protein folding	*HSPA4, HSPA4L*

## 9 Existing gaps and prospective directions for future research

The integration of genomics, transcriptomics, proteomics, and metabolomics data is crucial for understanding the complex regulatory networks that impact semen quality. By considering all of these factors together, we can uncover interactions between genes, proteins, and metabolites that are not evident when studying each omics layer independently. Although this review identifies many genetic markers associated with semen quality, it is important for future research to focus on the functional validation of these markers. Technologies like CRISPR-Cas9 and RNA interference (RNAi) can be utilized to confirm the roles of these genes in spermatogenesis and fertility. In addition to genetic markers, epigenetic modifications such as DNA methylation, histone modification, and non-coding RNAs may also play significant roles in semen quality. It is essential for future studies to explore how these epigenetic factors influence gene expression related to sperm function. By conducting comparative studies across different livestock species, we may be able to identify conserved genetic pathways and markers that are crucial for reproductive success. This would provide insights that can be applied across species. However, it is important to note that while the review identifies numerous genetic markers associated with semen quality, many of these markers have not been functionally validated. This limitation hinders the direct application of these findings in breeding programs. Furthermore, it is worth considering that the genetic markers identified are often specific to certain species, limiting their generalizability across different livestock species. This poses a challenge for developing universal breeding strategies. Semen quality is a multifactorial trait influenced by various genes, environmental factors, and their complex interactions. Due to the complexity of these interactions, it is difficult to identify single markers that can reliably predict fertility outcomes. Differences in breed, animal age, and health status are critical factors that significantly influence semen quality. These variables should be carefully considered in future research studies to ensure comprehensive and accurate findings.

## 10 Conclusion

In conclusion, the identification and understanding of genetic markers associated with semen quality traits in livestock have the potential to significantly enhance reproductive efficiency and genetic improvement in animal breeding programs. Thanks to advancements in genomic technologies and molecular biology, we can now pinpoint specific genes and genetic variations that impact semen quality, including sperm motility, concentration, morphology, and overall fertility. This knowledge is vital for developing targeted breeding strategies that aim to improve these traits, ultimately leading to enhanced reproductive outcomes and increased productivity in livestock populations. By incorporating genetic markers into selective breeding programs, livestock producers can achieve higher fertility rates, improve genetic diversity, and increase economic benefits. Future research should focus on validating these genetic markers across different breeds and environments to ensure their practical application in diverse farming systems. Ultimately, integrating genetic insights into reproductive management practices will play a crucial role in ensuring the sustainability and profitability of livestock industries worldwide. Furthermore, this review is based on data from various studies, but inconsistencies in study design, sample sizes, and analytical methods across studies can lead to conflicting results. This variability complicates the synthesis of findings and the identification of reliable markers.

## Glossary

CAPZA3: Capping actin protein of muscle Z-line subunit alpha 3
CAPZB: Capping actin protein of muscle Z-line subunit beta
CASP3: Caspase 3
CATSPER1: Cation channel sperm associated 1
CDH2: Cadherin 2
CDH1: Cadherin 1
TGFB2: Transforming growth factor beta 2
ACTB: Actin beta
ACTR2: Actin related protein 2
ADAD1: Adenosine deaminase domain containing 1
ATP1B1: ATPase Na+/K+ transporting subunit beta 1
CFAP58: Cilia and flagella associated protein 58
CRISP3: Cysteine-rich secretory protein 3
CROCC2: Ciliary rootlet coiled-coil, rootletin family member 2
CSNK1G2: Casein kinase 1 gamma 2
DAZL: Deleted in azoospermia like
DICER1: Dicer 1, ribonuclease III
DPY19L2: Dpy-19 like 2
ESR1: Estrogen receptor 1
FSHR: Follicle stimulating hormone receptor
GNRHR: Gonadotropin releasing hormone receptor
HOOK1: Hook microtubule tethering protein 1
FYN: FYN proto-oncogene, Src family tyrosine kinase
ICAM1: Intercellular adhesion molecule 1
HSPA4L: Heat shock protein family A (HSP70) member 4 like
INHA: Inhibin subunit alpha
INHBA: inhibin subunit beta A
INHBC: Inhibin subunit beta C
INHBE: Inhibin subunit beta E
JAM3: Junctional adhesion molecule 3
KIT: KIT proto-oncogene, receptor tyrosine kinase
KLHL10: Kelch like family member 10
MAP3K1: Mitogen-activated protein kinase 1
MDC1: Mediator of DNA damage checkpoint 1
MEIOC: Meiosis specific with coiled-coil domain
TEKT3: Tektin 3
TGFB2: Transforming growth factor beta 2
TGFBR1: Transforming growth factor beta receptor 1
TNF: Tumor necrosis factor
TNP1: Transition protein 1
TUBB: Tubulin beta class I
VCAM1: Vascular cell adhesion molecule 1
ZSWIM7: Zinc finger SWIM-type containing 7
TNP2: Transition protein 2
MOV10L1: Mov10 like RISC complex RNA helicase 1
MSH2: MutS homolog 2
MTNR1A: Melatonin receptor 1A
NOS2: Nitric oxide synthase 2
OAZ3: Ornithine decarboxylase antizyme 3
PPP1CC: Protein phosphatase 1 catalytic subunit gamma
PRKAR1B: Protein kinase cAMP-dependent type I regulatory subunit beta
PRLR: Prolactin receptor
PRM1: Protamine 1
RAB33B: RAB33B, member RAS oncogene family
RAD51B: RAD51 paralog B
SMAD3: SMAD family member 3
SPAG6: Sperm associated antigen 6
SRC: SRC proto-oncogene, non-receptor tyrosine kinase
STRA8: Stimulated by retinoic acid 8
TDRD5: Tudor domain containing 5
SUN5: Sad1 and UNC84 domain containing 5
SYCE3: Synaptonemal complex central element protein 3
